# Supplementary figures and images for: eGAC3D: enhancing depth adaptive convolution and depth estimation for monocular 3D object pose detection
Source: PeerJ Comput Sci. 2022 Nov 3;8:e1144. doi: 10.7717/peerj-cs.1144 (PMC9680869; doi:10.7717/peerj-cs.1144)

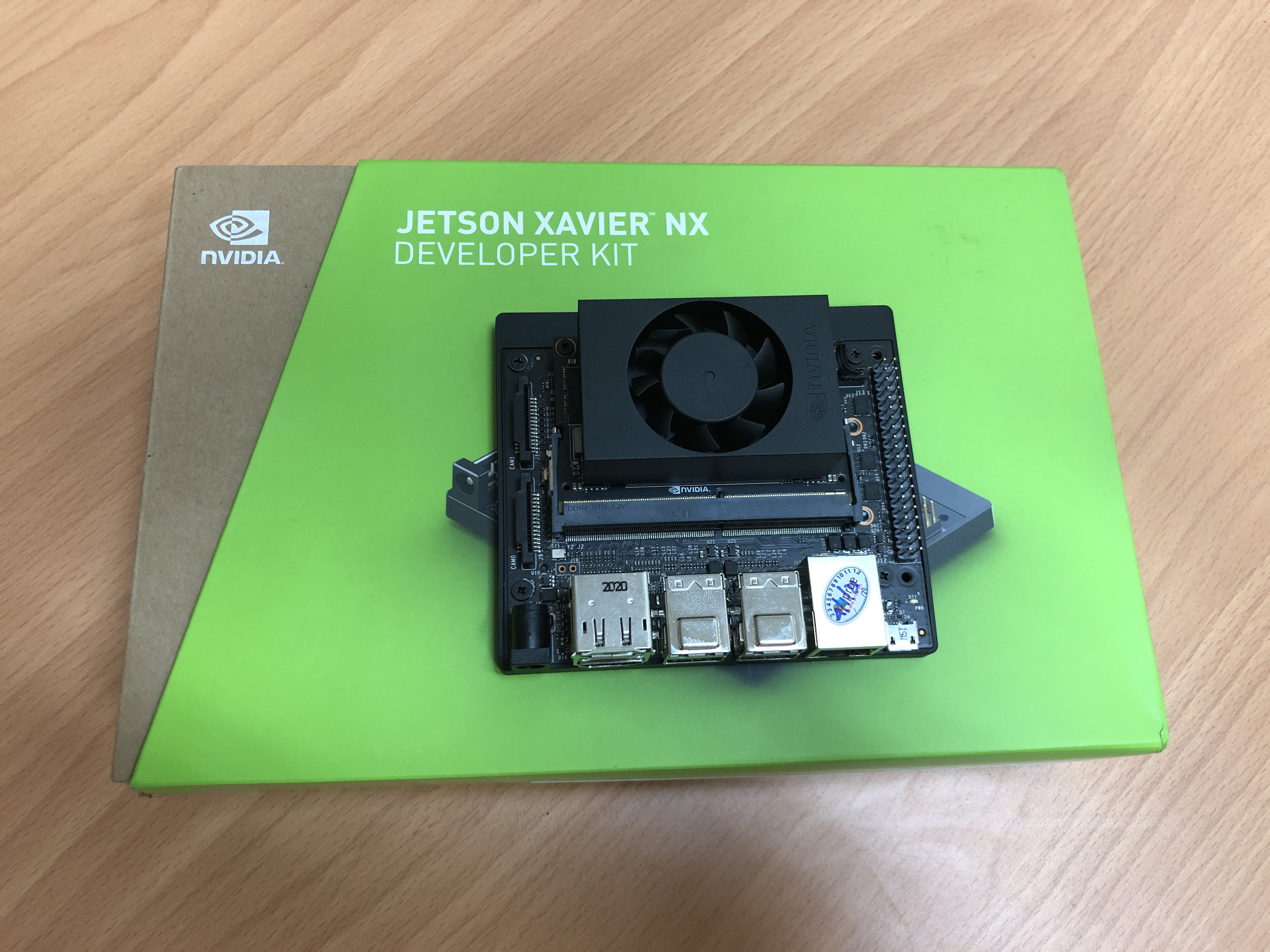

Supplement: Supplemental Information 1 [file peerj-cs-08-1144-s001.zip › eGAC3D-code/images/jetson_xaviernx.jpg]

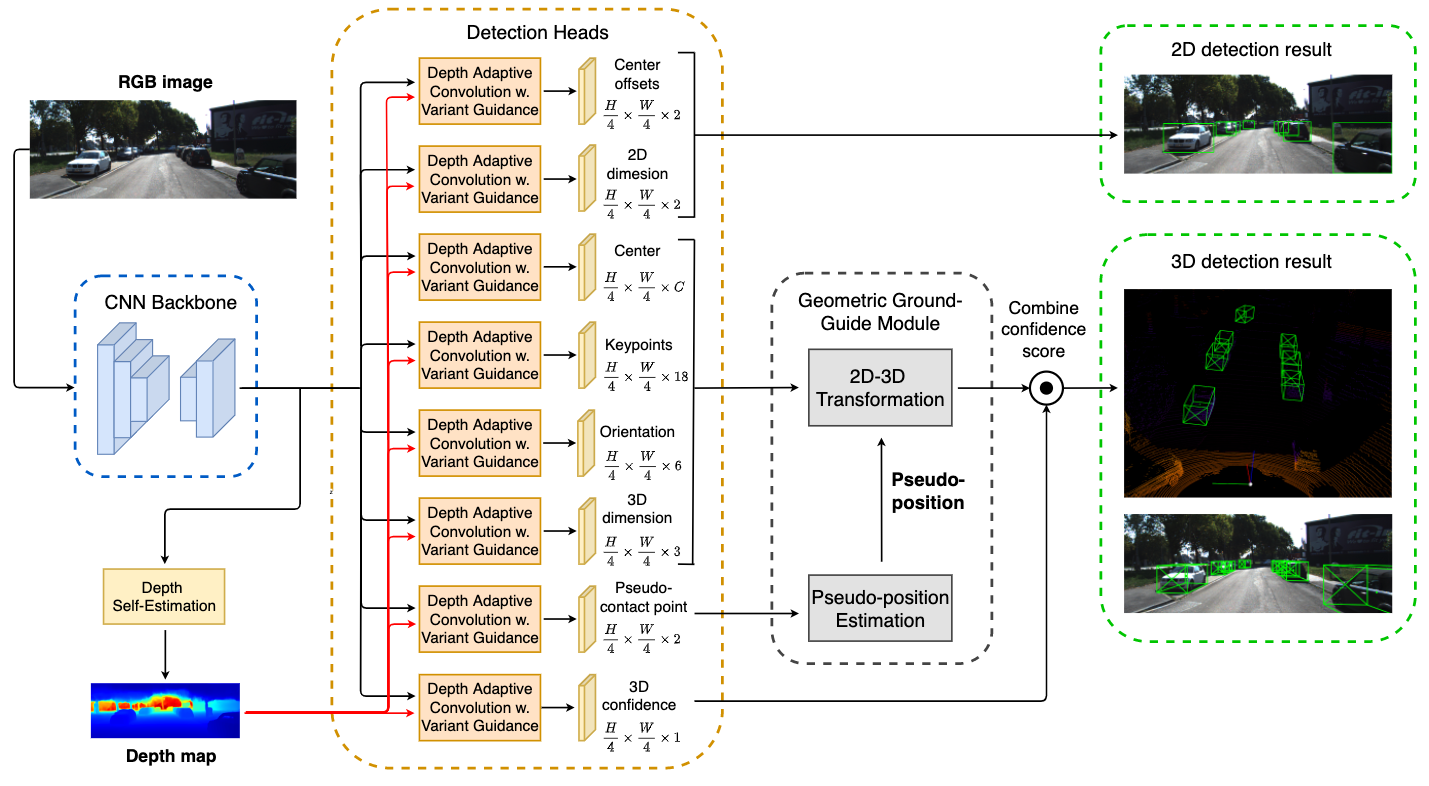

Supplement: Supplemental Information 1 [file peerj-cs-08-1144-s001.zip › eGAC3D-code/images/architecture.png]
